# Ultrafast Investigation of Intramolecular Charge Transfer and Solvation Dynamics of Tetrahydro[5]-helicene-Based Imide Derivatives

**DOI:** 10.1038/srep24313

**Published:** 2016-04-14

**Authors:** Huaning Zhu, Meng Li, Jiangpu Hu, Xian Wang, Jialong Jie, Qianjin Guo, Chuanfeng Chen, Andong Xia

**Affiliations:** 1 Key Laboratory of Photochemistry, Beijing National Laboratory for Molecular Sciences (BNLMS), Institute of Chemistry, Chinese Academy of Sciences, Beijing 100190, People’s Republic of China; 2 Key Laboratory of Molecular Recognition and Function, Beijing National Laboratory for Molecular Sciences (BNLMS), Institute of Chemistry, Chinese Academy of Sciences, Beijing 100190, People’s Republic of China

## Abstract

We report the excited-state intramolecular charge transfer (ICT) characteristics of four tetrahydro[5] helicene-based imide (THHBI) derivatives with various electron-donating substitutes in different polarity of solvents using steady-state, time-resolved transient absorption (TA) spectroscopy. It is found that, the small bathochromic-shift of the absorption spectra but large red shift of the emission spectra for all dyes with increasing solvent polarity indicates the larger dipole moment of the excited state compared to ground state. The results of theoretical calculations exhibit the charge transfer from the terminal donors to helical backbone, which accounts for the degrees of red shift of the emission spectra from different extent of ICT nature. Time-resolved TA spectra recorded as a function of electron-donating substitutes and solvent polarity show the dye with stronger donors (THHBI-PhNPh_2_) in more polar solvent behaves faster excited-state ICT relaxation, leading to the formation of solvent-stabilized ICT state (ICT’ state) from the excited ICT state; The dyes (THHBI-Ph, THHBI-PhCF_3_ and THHBI-PhOMe) with relative weaker donors show weaker dependence on solvent polarity, and instead of that intersystem crossing (ISC) becomes possible from ICT state to triplet state.

Organic molecular materials with electronic push-pull properties have attracted considerable attention because their photophysical properties based on intramolecular charge transfer (ICT) could be widely tuned through appropriate modification of the chemical structures of donor, acceptor substitutes and/or the π-conjugated linkers for practical applications^1^,^2^,^3^,^4^,^5^,^6^. The photophysical properties, such as fluorescence quantum yield, fluorescence lifetime, especially the band gap of these organic molecules are strongly dependent on the ICT characteristic from different push-pull abilities between donor and acceptor^7^,^8^,^9^,^10^. Obviously, the chemical structures and surrounding environments could be the most critical factors for their practical behaviors^11^,^12^,^13^,^14^,^15^. In order to acquire excellent photophysical properties, a lot of strategies including enhanced intramolecular charge transfer, introduction of different substituents and modification of molecular structures have been developed^8^,^10^,^14^,^16^,^17^,^18^. Recently, we have found that the helicene derivatives showed good spectral properties with intense fluorescence in both solution and solid state^16^,^19^.

Helicene derivatives have draw great attention because of their applications such as ligands in asymmetric catalysis^20^, chiral discotic liquid crystalline component^21^, second-order nonlinear optical properties^17^ and enantioselective fluorescent sensors^22^. The helicene derivatives, a class of polycyclic arenes with nonplanar and rigid configuration, are expected to improve the fluorescence quantum yield compared to planar and nonrigid conjugation core because of the nonplanar structure. The interplanar angles (or the dihedral angles) of the two terminal rings can be modulated with the lengths of the helicenes and the substituents, so that the intermolecular π-π stacking and aggregation could be avoided^16^,^18^. Meanwhile, the benzene ring of the helicene core could extend the π-conjugated geometry when the electron-donating groups including phenyl groups is introduced to the terminal of the helicene core, which would increase the charge transfer. Thus, the push-pull electronic effect, conjugative effect and the twisted structure in these dyes could greatly affect the ICT characteristic.

In this article, we report a new kind of organic dyes with ICT characteristic based on tetrahydro[5] helicene core (the molecular structures are shown in Fig. 1)^19^. The aim of this work is to explore whether ICT properties are improved by increasing the electron-donating abilities of donor based on helicene, for which, we performed a comparative study of the dyes containing the nonplanar tetrahydro[5] helicene-based imide (THHBI) as the central acceptor with different donors (donor strength: THHBI-PhCF_3_: p-trifluoromethylphenyl <THHBI-Ph: phenyl <THHBI-PhOMe: p-methoxyphenyl <THHBI-PhNPh_2_: triphenylamine). The surrounding solvent is known to have significantly influence on the ICT properties, thus the spectral properties in different solvents were further investigated. Time-resolved transient absorption spectra show that the THHBI-PhNPh_2_ with stronger donor in more polar solvent behaves faster excited-state ICT relaxation, leading to the formation of ICT state and further solvent-stabilized ICT state (ICT’ state); The THHBI-Ph, THHBI-PhCF_3_ and THHBI-PhOMe with relative weaker donors show weaker dependence on the solvent polarity, and then leading to enhance intersystem crossing (ISC) from excited ICT state to triplet state. The larger bathochromic shift of fluorescence spectra than that of the absorption spectra for all dyes in different solvents means the larger ICT characteristic and dipole moment of excited state than that of ground state, suggesting that the quantum yield and fluorescence lifetime could be modulated by the different electron-donating abilities of donor groups and solvent polarity. Furthermore, the theoretical calculations clearly display the charge redistribution from donor moieties to helicene-based imide acceptor in excited state compared with ground state. The studies presented here provide not only a fundamental understanding of the structure-property relationships and solvent-dependent photophysical performances but also a guide for designing new compounds with favorable CT properties for applications as materials in electroluminescence devices, solar cells and thin film transistors^3^,^4^,^23^.

## Results and Discussion

### Steady state spectral characteristics and DFT calculations

**Figure 2:**
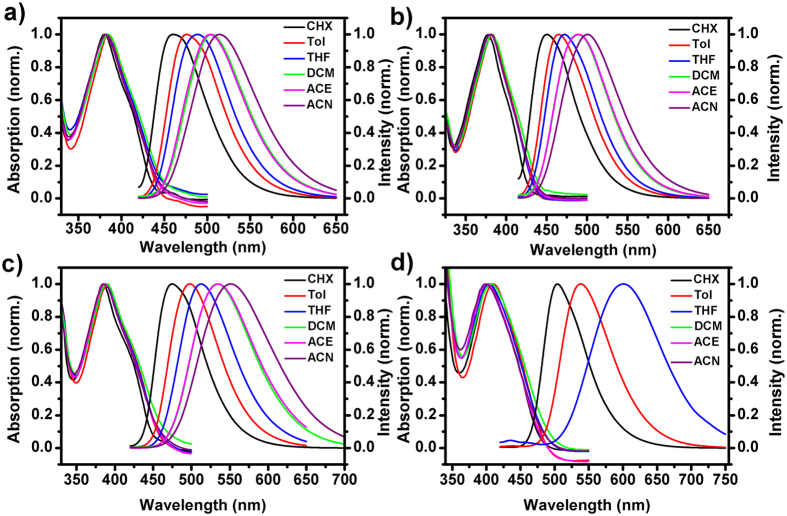
Normalized absorption and emission spectra of the tetrahydro[5] helicene-based imide dyes in different solvents. (**a**–**d**) are for THHBI-Ph, THHBI-PhCF_3_, THHBI-PhOMe and THHBI-PhNPh_2_, respectively.

**Figure 3:**
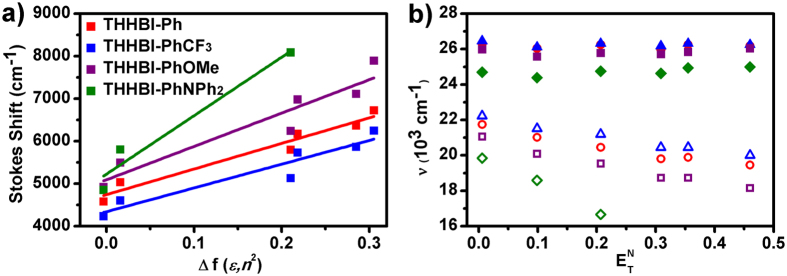
(**a**) Plots of Stokes shifts as a function of the solvent polarity parameter Δ*f* (*ε*, *n*^2^) for all dyes in selected solvents. Slope (cm^−1^): THHBI-Ph: 6028 ± 700; THHBI-PhCF_3_: 5591 ± 900; THHBI-PhOMe: 7851 ± 1300; THHBI-PhNPh_2_: 13762 ± 2900. (**b**) Absorption (filled) and emission (open) peaks against the solvent polarity (expressed in the form of the Reichhardt parameters). THHBI-Ph (○), THHBI-PhCF_3_ (Δ), THHBI-PhOMe (◻) and THHBI-PhNPh_2_ (◊).

Figure 2 shows the normalized optical absorption and emission spectra of all investigated helicene-based imide dyes in different solvents. The solvents including cyclohexane (CHX), toluene (Tol), tetrahydrofuran (THF), dichloromethane (DCM), acetone (ACE) and acetonitrile (ACN) were used in the measurements. All dyes display intense absorption in the UV range with peak around 380 nm for THHBI-Ph and THHBI-PhCF_3_, 390 nm for THHBI-PhOMe, and 400 nm for THHBI-PhNPh_2_ in various polarities of solvents, respectively. All the absorption maxima displayed by THHBI derivatives in various solvent are ascribed to the charge-transfer states, which can be further proved by following quantum chemical calculations results. It is found that the absorption maximum for each dye shows nearly no noticeable shift but the emission spectra show appreciable bathochromic shift with increase in polarity of solvents, indicating nearly zero dipole moment in the ground state but large dipole moments in the excited state^24^,^25^,^26^. The peak positions of absorption and emission spectra for all dyes as a function of the solvent polarity are displayed in Fig. 3b. It is clearly shown that the solvatochromism is far more obvious in emission than that in absorption. In the same solvent, it’s worth noting that the extent of the bathochromic shift of different dyes is also different (Supplementary Table S1) where the descending order of the red shift of fluorescence spectra for THHBI-PhNPh_2_, THHBI-PhOMe, THHBI-Ph and THHBI-PhCF_3_ are observed. This positive solvatochromism phenomenon could be caused by the intramolecular charge transfer process^11^,^27^,^28^,^29^,^30^. The fluorescence lifetime and quantum yield are shown in Tables S5 and S6 of Supplementary Information. The Fluorescence decay profiles are shown in Fig. S1. It is found that for THHBI-Ph and THHBI-PhCF_3_, the lifetime increases with the enhancement of the solvent polarity. THHBI-PhOMe also shows this trend except that in ACE and ACN which are still longer than that in the low polar solvent, i.e., THF, Tol, CHX. THHBI-PhNPh_2_ has longer lifetime in low polar solvents and shorter one in strong polar solvents. Furthermore, it is found that the quantum yields firstly rise then descend with increasing solvent polarity for THHBI-Ph, THHBI-PhCF_3_ and THHBI-PhOMe. While the quantum yield of THHBI-PhNPh_2_ decreases with the increase of the solvent polarity even quenching in more polar solvents such as DCM, ACE, ACN. Usually, the chromophores with ICT characteristic interact strongly with polar solvent, which would enhance the nonradiative decay rate and result in lower quantum yield^11^,^31^,^32^, THHBI-PhNPh_2_ is consistent with this case. It is wondering that, why the other three dyes show the abnormal change with increasing solvent polarity? The answer may be due to the molecular torsion. In fact, upon excitation, the size and direction of the dipole moment of the chromophore with ICT characteristic are changed from ground state to excited state. Then the solvent molecule around the chromophore would reorientate to reach a new equilibrium state. In more polar solvent, more solvent molecules around the chromophore could hinder the torsion of the chromophore, which decrease the energy dissipation to the surrounding environment. However, this mechanism can not interpret the positive relation of the quantum yield with the solvent polarity of THHBI-PhNPh_2_, the ISC process could be the possible pathway competing with the radiation. To identify this processes, we performed the laser flash photolysis experiment in various gas condition (shown in Supplementary Information), which proves the existence of the triplet state in THHBI-Ph, THHBI-PhCF_3_ and THHBI-PhOMe but not in THHBI-PhNPh_2_. In different polarity of solvents, the energy level of the S_1_ state is modulated by the solvent polarity, which can be reflected from the emission spectra. The lower energy level of S_1_ state in more polar solvent would make ISC difficult to happen, then the radiation transition is the main way for decaying back to ground state. Similar cases were reported previously^33^,^34^. When the solvent polarity increases such as ACE, ACN, the nonradiation processes play a dominant role in decrease the quantum yield.

To evaluate the ICT characteristic, we have carried out the DFT and TD-DFT calculations. It is found that the calculated energy gap (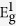) between HOMO (highest occupied molecular orbital) and LUMO (lowest unoccupied molecular orbital) and energy level of S_1_ (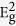) for all dyes are very close to the experimental values (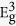) in CHX (Supplementary Table S3). This proves the transitions with ICT characteristic mainly happen between HOMO and LUMO. Table S2 shows that the electronic density at the helicene-imide backbone significantly increases accompanying with a decrease at the lateral donor groups from HOMO to LUMO, which provides a clear picture of the ICT characteristic^35^. The CDD (charge difference density, shown in Supplementary Table S2) further shows the same ICT nature as above. When comparing the CDD and the electron density distribution in the HOMO and LUMO for different dyes, one can discover that the extent of the charge transfer is clearly different from each other. The THHBI-PhNPh_2_ possesses the largest ICT characteristic, in which the charge almost completely transfers from donors to the acceptor. THHBI-PhCF_3_ has the smallest charge redistribution. And THHBI-PhOMe and THHBI-Ph are in the medium strength. This is consistent with the degree of Stokes shifts and change of the dipole moments (below) between ground state and excited state.

### Estimation of dipole moment

The ICT nature of electronic push-pull molecules with dipole moments and dipole moment change between ground and excited state is typically dependent on their molecular structures. To investigate the ICT characteristic caused by the structures and solvent polarity, we performed the dipole moment estimation by measuring the relationship between the Stokes shift and solvent polarity from Lippert-Mataga equation (Equation 1)^36^,^37^. Similar studies of the photophysical properties dependence on solvents are found for many other donor-acceptor systems^11^,^12^,^27^,^31^,^32^. This method provides an estimation of the difference of dipole moments between the excited and ground state (Δ*μ*_*eg*_).


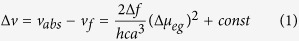


where Δν is the Stokes shift in a given solvent, ν_*abs*_ and ν_*f*_ are the wavenumbers of absorption and emission maxima, respectively, *h* is Planck’s constant, *c* is the velocity of light in vacuum, *a* is the Onsager cavity radius, *ε* and *n* are the dielectric constant and refractive index of solvent, respectively. 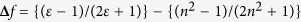 is the polarity function. Figure 3a shows the Δν vs Δ*f* plots for all dyes. The Onsager cavity radii and ground state dipole moments (*μ*_*g*_) are obtained from quantum chemical calculations. Finally, the excited state dipole moment (*μ*_*e*_) and Δ*μ*_*eg*_ (Supplementary Table S4) could be calculated from Equation (1). It is found that, the Δ*μ*_*eg*_ is almost the same for THHBI-Ph and THHBI-PhCF_3_ (about 13.0 D), and the Δ*μ*_*eg*_ value are found to be about 15.3 D and 24.3 D for THHBI-PhOMe and THHBI-PhNPh_2_, respectively, which are agreement with the observed spectral shifts as mentioned above. Meanwhile, the excited state dipole moments are also obtained with the strengths in the order: THHBI-PhNPh_2_ >THHBI-PhOMe >THHBI-Ph >THHBI-PhCF_3_. This is closely related to the different electron-donating abilities of donors, i.e., triphenylamine >p-methoxyphenyl >phenyl >p-trifluoromethylphenyl. The fact that the dipole moments of ground states for all dyes are very small less than 5.1 D, indicates that the large changes of dipole moment between ground state and excited state mainly result from excited state charge redistribution, reflecting more polar characteristic of S_1_ state than ground state. The various polarity of S_1_ state would have prominent effect on excited-state relaxation dynamics, which reflects the extent of ICT as well. To elucidate solvent dependent excited state manifold, time-resolved transient absorption spectroscopy was further need.

### Time-resolved transient absorption spectra

The steady-state spectral results and dipole moment estimation show the different extent of ICT distribution and various dipole moment changes of the dyes upon excitation, which could lead to different luminescence properties and the excited state solvation dynamics. To fully explore the role of the ICT, the time-resolved transient absorption (TA) spectroscopy was performed^12^,^38^,^39^,^40^,^41^.

### Excited state dynamics of THHBI-PhOMe

**Figure 4:**
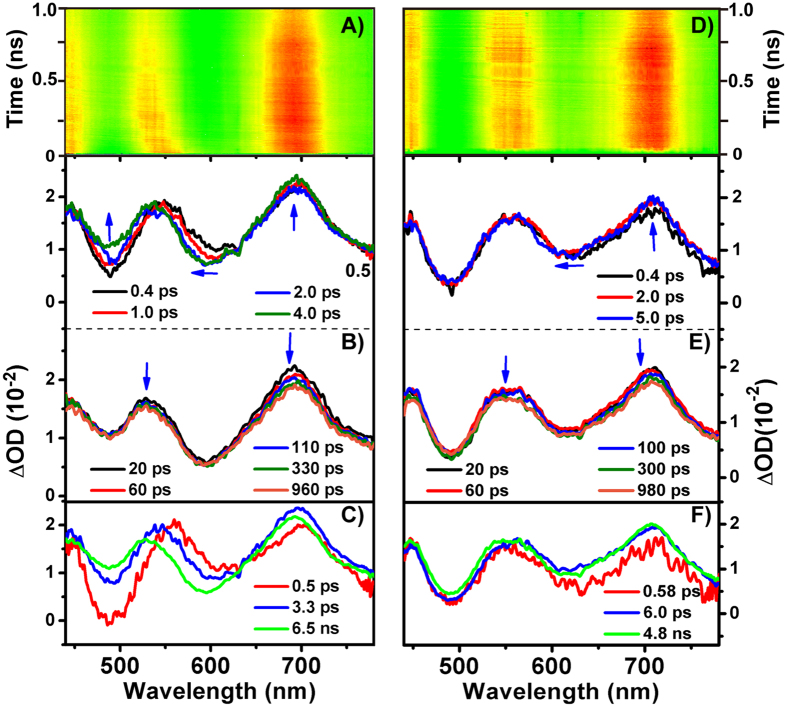
The transient absorption spectra of THHBI-PhOMe in THF (**A**–**C**) and Tol (**D**–**F**) after 400 nm excitation. (**A**,**D**) contour plot of the experimental data. (**B**,**E**) evolution of the time-resolved spectra. (**C**,**F**) EADS obtained through global fitting. Kinetics at selected single wavelengths for showing the quality of the global fitting are shown in Supplementary Fig. S6a.

Figure 4A,D give a contour plot of the experimental data in THF and toluene, respectively. It is found that the TA spectra of THHBI-PhOMe in THF are composed of a broad excited state absorption (ESA) with two peaks around 700 nm and 550 nm overlapping with the stimulated emission (SE) between the 450 nm and 525 nm (Fig. 4B). After about ~4 ps, the 700 nm ESA peak gradually shifts to 690 nm and the middle ESA peak moves from 550 nm to 530 nm. Simultaneously, the SE band rises because of the overlapping with blue-shift of the original middle ESA which are reflected by the kinetic traces at 565 nm and 493 nm (Supplementary Fig. S4c). After long time, the spectra decay with the middle ESA slight blue shift. In toluene, the TA spectra are almost the same as that in THF at the initial time (Fig. 4E). However, the two ESA peaks show smaller blue-shift (several nanometers) in contrast to that (tens nanometers) in THF with the increase of time delays. Different extent of blue shift of ESA in these two different polar solvents suggests that the ICT are significantly affected by the solvent polarity, which is in concert with previous reports^27^,^42^,^43^. The more polar solvents could accelerate the relaxation processes and give rise to the larger hypochromatic shift until solvation is finished. Similar femtosecond TA spectra and the dynamics are also found for THHBI-Ph, and THHBI-PhCF_3_, which are introduced in S4 section of Supplementary Information.

**Figure 5:**
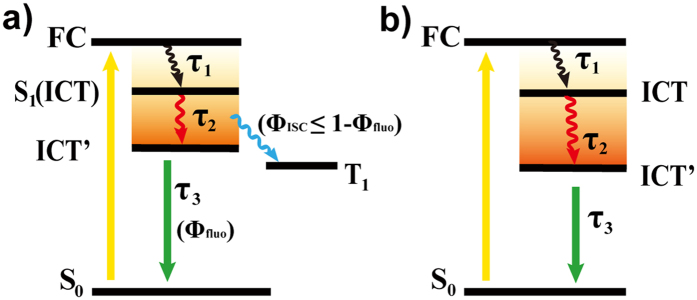
Relaxation pathways of the helicene-based imide derivatives based on global fitting. (**a**) THHBI-Ph, THHBI-PhCF_3_, THHBI-PhOMe and (**b**) THHBI-PhNPh_2_. Φ_fluo_ and Φ_ISC_ are the fluorescence and ISC quantum yield.

**Figure 6:**
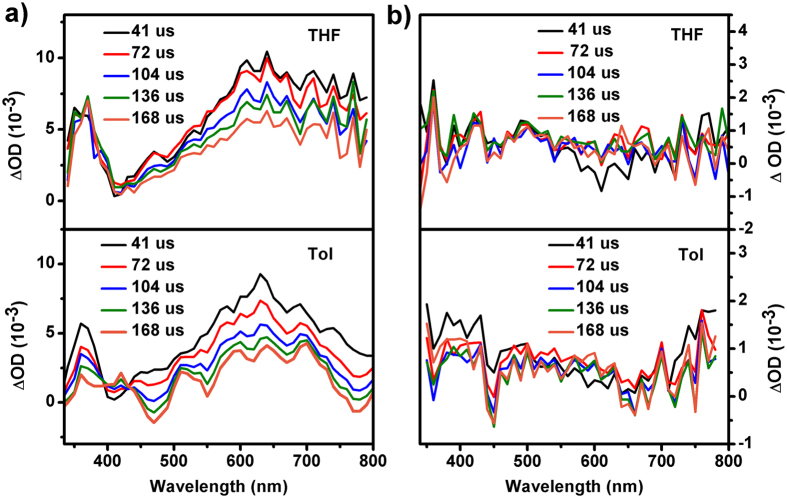
The nanosecond transient absorption spectra of THHBI-PhOMe (**a**) and THHBI-PhNPh_2_ (**b**) in N_2_ purged solutions. The spectra in air condition is shown in Supplementary Fig. S11.

Global analysis with a sequential model (Fig. 5a) gives three components and the evolution-associated difference spectra (EADS) are shown in Fig. 4C,F. The fast component of ~0.5 ps in THF (~0.55 ps in Tol, Table 1) are the relaxation time from the Frank-Condon (FC) to the solvent related ICT state. The second components are assigned to the ICT → ICT’ state. The two time constants are longer than the solvation time of THF (0.94 ps)^44^ and Tol (2.4 ps)^45^, respectively. Thus, the relaxation could be due to the solvation and vibrational relaxation of the excited state along with the conformation rearrangement^27^,^28^,^46^. This solvent-coupled excited state relaxation is considerable faster in THF than that in toluene, indicating that the interaction between the THHBI-PhOMe molecule with ICT nature and THF molecule is stronger identified by the larger ESA blue shift. The slow component in nanosecond scale is the decay of S_1_ state. From the EADS we also see the larger shift of ESA in THF than in Tol. To figure out the luminous mechanism, the laser flash photolysis experiments were carried out providing the appearance of the triplet state similar with THHBI-Ph and THHBI-PhCF_3_ in both solvents, and the lifetimes under different surrounding environments are shown in Table S8. It suggests at least two mainly relaxation pathways of S_1_ state: one is radiative transition to ground state and the other is intersystem crossing to T_1_ state (Fig. 6a, Supplementary Fig. S11). Thus, ISC is a competing pathway of affecting the fluorescence quantum yield.

### Excited state dynamics of THHBI-PhNPh_2_

**Figure 7:**
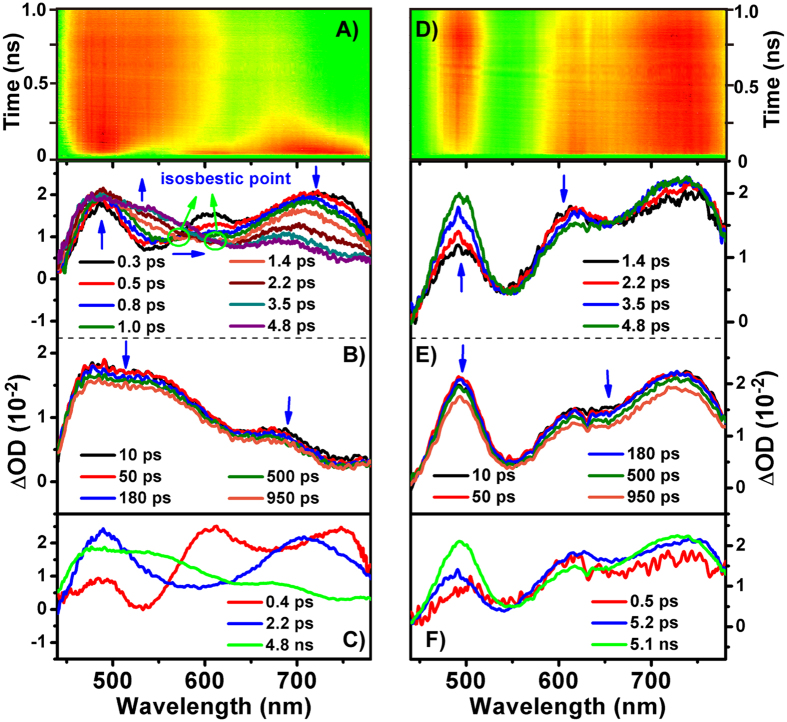
The transient absorption spectra of THHBI-PhNPh_2_ in THF (**A**–**C**) and Tol (**D**–**F**) after 400 nm excitation. (**A**,**D**) contour plot of the experimental data. (**B**,**E**) evolution of the time-resolved spectra. (**C**,**F**) EADS obtained through global fitting. Kinetics at selected single wavelengths for showing the quality of the global fitting are shown in Supplementary Fig. S6b.

Compared with THHBI-Ph, THHBI-PhCF_3_ and THHBI-PhOMe, different excited state features were obtained for THHBI-PhNPh_2_. Figure 7A,D show the contour plot of the experimental data. In THF, we can see a broad ESA band with three maximums around 735 nm, 610 nm and 490 nm overlapped with the SE band around 550 nm upon excitation (Fig. 7B). In about 1.4 ps, the 735 nm band decreases and shifts to 707 nm, while the 490 nm band rises and the 610 nm peak decays. Meanwhile, the SE around 550 nm displays significant red shift from 535 nm to 580 nm with a clear isosbestic point at 570 nm. This evolution corresponds to the conversion from FC state to solvent related ICT state. The decay of the 610 nm peak is due to the rise of the 490 nm ESA band and the red shift of SE band, which could be clearly seen from the kinetic traces at 500 nm and 610 nm (Supplementary Fig. S3a). With increased time delays to 5 ps, the original ESA band around 735 nm continues to decrease and shift to 700 nm whose decay accompany with the rise of the 550 nm band (Supplementary Fig. S4a). Meanwhile, the SE band keeps on red shifting. The isosbestic point appearing at 610 nm indicates the spectral conversion from ICT to conformationally relaxed ICT state (ICT’ state). Global analysis with the sequential model (Fig. 5b) gives three time constants (0.4 ps, 2.2 ps and 4.8 ns) and corresponding EADSs are displayed in Fig. 7C. Considering the above analysis, the first ultrafast component is the FC → ICT conversion, and the second one (2.2 ps) belongs to the evolution from ICT to ICT’ state. The last time constant is the lifetime of the lowest excited state. In Tol, the TA spectrum is the same as that in THF after initial excitation (Fig. 7E). With increased time delays, the 735 nm ESA displays little rise and blue shift (several nanometers) while the 610 nm band decays. The 490 nm ESA band also increases accompany with the SE band shifting from 540 nm to 549 nm. The SVD and global fitting also give three time constants: 0.5 ps, 5.2 ps and 5.1 ns (Fig. 7F), which are ascribed to the relaxation FC → ICT, ICT → ICT’ and the decay of S_1_ state, respectively. The initial fast spectral evolution is ascribed to a fast vibrational relaxation within the excited state^31^,^47^. The faster ICT-ICT’ process in THF compared to toluene indicates it is affected by solvent polarity, which could be come from the solvation accompanying with the conformation rearrangement. To interpret solvent-related radiation pathway, the laser flash photolysis experiments were performed to show no triplet states appearance of THHBI-PhNPh_2_ (Fig. 6b, Supplementary Fig. S11). Therefore, the relaxation of S_1_ state is directly through the radiative and nonradiative transition to ground state. The nonradiative transition is the primary pathway of decreasing the fluorescence quantum yield.

Taking a closer look at Fig. 7, we found that the TA spectra are the same in two solvents after the initial excitation. However, with increasing time delays, the spectral evolution is different from each other. In THF, the spectra clearly show three stages of the blue shift of ESA and red shift of SE divided by two isosbestic points, which corresponds to the three dynamical relaxation processes. While in Tol, not only the smaller spectral shift of ESA and SE band than that of THF but also no clear stages and isosbestic points are observed. It is clear that the dynamics are apparently faster in THF. Considering the above conclusions from the steady state spectroscopy and quantum chemical calculations that the THHBI-PhNPh_2_ has the largest ICT characteristic, THHBI-PhNPh_2_ molecules have strongest interaction with more polar THF molecules. Therefore, these different spectral characteristics are significantly caused by the inherent molecular structure and affected by solvent polarity.

**Figure 8:**
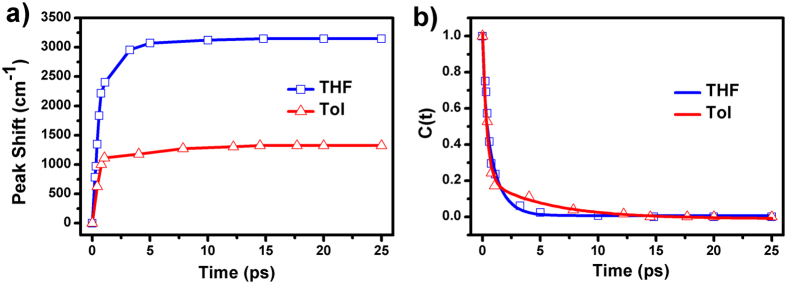
(**a**) Peak shift dynamics; (**b**) excited-state peak shift time correlation functions (C(t)) and fits of THHBI-PhNPh_2_. The average time is 0.8 ps for THF and 1.45 ps for Tol, respectively.

However, the dynamics of red shift of SE peak of THHBI-PhNPh_2_ in femtosecond TA spectra could results from both vibrational cooling and solvation. To figure out the reason, a solvation correlation function is calculated by^43^,^44^,^48^,^49^,^50^:


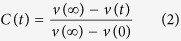


where *v*(*t*) is the position of SE peak at a delay time *t*, and the *v*(0) and *v*(∞) denote the initial and final positions of the peak after photoexcitation, respectively. It is difficult to determine the *v*(0) directly owing to limited time resolution. Instead, the emission peak in non-polar CHX is used as approximation to *v*(0) as the Fee-Maroncelli procedure^51^ while *v*(∞) is fixed at the final peak position in each solvent. The TA spectra at different time delays were fitted with Gaussian functions (see Supplementary Fig. S7). The time dependent peak shift can be calculated from the fitted Gaussian peak position at different time delays. The peak shift dynamics and corresponding correlation functions are displayed in Fig. 8. The correlation functions of THHBI-PhNPh_2_ in THF and Tol are fitted well with two exponentials which are summarized in Supplementary Table S7. The average time constants (0.8 ps for THF and 1.45 ps for Tol) are in well accord with the average solvation times reported by Maroncelli and co-workers^44^. The first ultrafast solvation time is commonly considered as the inertial relaxation, and the slower solvation component is due to diffusional or conformation reorganization of the solvent molecules around the excited sample^27^,^44^. These conclude that the significant spectral dynamics of THHBI-PhNPh_2_ mostly originate from solvation. The peak shift dynamics suggests solvation in THF is much faster and more prominent than that in Tol.

### Comparison of the ICT dynamics

As above description, the helicene-based imide organic dyes display various excited state dynamics in different polarity of solvents, which suggests that the photophysical properties of dyes are sensitive to the surrounding environments. On the other hand, the organic dye molecules with distinct structures exhibit different excited state relaxation processes in the same solvent. In both solvents, the same conclusions are obtained for all studied dyes. In the following, the THF solutions of these dyes are taken as a sample for discussion.

From the fitted parameters (Table 1), the first initial processes are on a scale of hundreds of femtoseconds for all chromophores, which are the internal conversion and/or vibrational relaxation of higher excited-state^31^,^47^. For the process from the ICT to ICT’ state, the spectral evolution of THHBI-PhNPh_2_ is the fastest and that of THHBI-PhCF_3_ is the slowest, THHBI-Ph and THHBI-PhOMe are in the medium. This trend is consistent with the extent of spectral shift of the TA data. This is a solvent-coupled excited state conformational and vibrational relaxation process, and closely related to dipole moment of the excited state typically caused by ICT. The fastest evolution of the THHBI-PhNPh_2_ indicates strongest solvent reorganization, that is, the largest changes of the dipole moments and degree of charge transfer between the ground and excited state. The ultrafast process of the THHBI-PhNPh_2_ mainly comes from the solvation with geometry rearrangement, identified by the solvation correlation function. While less solvation related spectral shift and slow excited state relaxation dynamics of the THHBI-PhCF_3_ means the small excited dipole moment and charge redistribution. THHBI-Ph and THHBI-PhOMe are in the medium case. The timescale of the solvent-related excited relaxation reflect the degree of the charge transfer inside the chromophores, which is in accord with theoretical calculations and steady state spectral experiments.

In addition, from the optimized geometry (shown in Supplementary Fig. S12 and Table S9), both the ground state and excited-state structures are twisted conformations owing to the nonplanar and rigid helicene group. The dihedral angle (*β* for C5-C6-C7-C8) between the donor group and the twisted helicene body becomes smaller in the excited-state than that of ground state, which suggests the relative planar conformation in the excited-state. It is owing to the tendency to form the planar conformation between the two side donors and helicene-based acceptor that makes larger torsion angle (*α* for C1-C2-C3-C4) of the non-planar helicene-based acceptor in excited state. This is consistent with previous report that the molecular structures are tend to form a relative planar conformation in the excited-state compared with the ground state upon photoexcitation^28^,^52^. In the ultrafast conversion process between the FC state and ICT state, the charge transfers from donor to the acceptor while the molecule still remains the relative planar conformation. For the THHBI-Ph, THHBI-PhCF_3_ and THHBI-PhOMe with less ICT characteristic, the solvation is relative weaker, the process from ICT to ICT’ state is slower with the conformational relaxation and along with ISC to triplet state, then decays to the ground state through radiative transition. However, in THHBI-PhNPh_2_, the ICT state is also a relative planar charge-transfer state, but the process from ICT state to the ICT’ state is much faster accompanied with the conformational rearrangement due to stronger interaction with the solvent molecule result from its large dipole moment and degree of the ICT nature. This ICT’ state is a twisted intramolecular charge transfer state, which may be close to the conformation of the ground state. During this solvation process, a portion of excitation energy dissipates into the surrounding environment. The nonradiative relaxation processes become increasingly efficient with the decreased energy gap between the ICT’ state and ground state. This is why the decline of the fluorescence yield and no appearance of ISC process in THHBI-PhNPh_2_. This process could be more remarkable with the increase of the solvent polarity^11^,^31^,^53^.

Furthermore, the different spectral behaviors could be related to the structural constitution. The helicene-based imide dyes have the same electron-withdrawing group (helicene-based imide group) with different electron-donating substituents. The electron-donating substituents of THHBI-Ph are phenyls, which extends the conjugation with the helicene backbone and results in the weak ICT characteristic. THHBI-PhCF_3_ has two p-trifluoromethylphenyl groups as donors. The Fluoride anion is the strong electron-withdrawing group, which can weaken the ICT from donor to helicene acceptor. Thus, the ICT characteristic is weaker in THHBI-PhCF_3_ than THHBI-Ph. In THHBI-PhOMe, two p-methoxyphenyl groups as electron-donors are linked with the helicene-based imide group. Generally, the methoxyl group has both inductive electron-withdrawing effect and the conjugated electron-donating effect. When the methoxyl is linked with phenyl, the lone pair electrons of oxygen atom are delocalized through conjugation with the phenyl group. Thus, the conjugated electron-donating effect plays a main role in THHBI-PhOMe and makes the donor stronger electron-push ability, which leads to the more intense ICT characteristic than THHBI-Ph. The donor groups of THHBI-PhNPh_2_ are two triphenylamines. The super-conjugated structure formatted by the lone pair of the central nitrogen atom with the linked phenyl groups in triphenylamine gives rise to the strongest electron-donating ability^6^,^31^,^32^,^54^. Therefore, it is no surprising to see that the strongest charge transfer from triphenylamine to the helicene acceptor happens in THHBI-PhNPh_2_. Additionally, the degree of the ICT characteristic of these molecules is also embodied in the bond length of the C-C bond linking the donor and acceptor groups. A decrease of the C-C bond length from 1.485 Å to 1.482 Å for increased electron-donating ability are observed in the examination of the results of geometry optimization (Supplementary Table S3), which reflects the degree of the charge transfer from the point of the molecular structures.

## Conclusions

The extent of intramolecular charge transfer and excited-state solvation dynamics of four different tetrahydro[5] helicene-based imide dyes are comparatively investigated by the experimental and theoretical methods. The large increase of the dipole moments after excitation is due to the ICT characteristic of the emissive state as reflected in the enhancements of the Stokes shift with increasing solvent polarity for all dyes, which is further supported by the theoretical calculations results that the charge transfer from different electron-donating groups to helicene-imide group. Using time-resolved transient absorption spectroscopy combined with global analysis, we have drawn a different picture of the excited state dynamics of the investigated dyes. For THHBI-Ph, THHBI-PhCF_3_ and THHBI-PhOMe with weak donors, the excited state decay through FC → ICT → ICT’ relaxation accompany with ISC, then the excitation energy deactivates by means of two competitive ways: radiative transition and intersystem crossing. The fluorescence quantum yields and lifetimes of THHBI-Ph, THHBI-PhCF_3_ and THHBI-PhOMe in different solvents are likely the consequence of their modulation. For THHBI-PhNPh_2_ with strong donor, the excited state relaxation pathway following a cascade model: FC → ICT → ICT’. The decay of its ICT state dominated through conformationally relaxed ICT’ state without the ISC.

The nonplanar screw-shaped skeletons and rigid configuration of the helicene derivatives make them difficult to form intermolecular π-π stacking and aggregates which will cause the fluorescence quenching. Thus, such nonplanar helicenes behave intense fluorescence even in high concentration solution or in the solid states. The non-planar packing such as herringbone-like pattern may be conducive to increase the intermolecular charge transfer distance which further generates long-lived charge-separated state and results in lower probability of geminate charge recombination^16^. Both the charge transfer efficiency and the photovoltaic conversion efficiency would be improved. The results presented in this work that the electron-donating ability and solvent polarity have significantly impact on the photophysical performances of the electronic push-pull helicene-based imide dyes provide a fundamental understanding of the structure-property relationships of the nonplanar helicenes. The D-A architectures based on helicenes with nonplanar screw-shaped configuration and prominent ICT characteristic could serve as potential scaffold for light harvesting and photovoltaic devices.

## Materials and Methods

### Materials

The synthesis and characterization of the tetrahydro[5] helicene-based imide dyes by Chen and co-workers was reported previously^19^. 2-dodecyl-8,11-dimethoxy-7,12-diphenyl-4,5,14,15-tetrahydro-1H-dinaphtho[2,1-e:1′,2′-g]isoindole-1,3(2H)-dione (THHBI-Ph). 2-dodecyl-8,11-dimethoxy-7,12-bis(4-(trifluoromethyl)phenyl)-4,5,14,15-tetrahydro-1H-dinaphtho[2,1-e:1′,2′-g]isoindole-1,3(2H)-dione (THHBI-PhCF_3_). 2-dodecyl-8,11-dimethoxy-7,12-bis(4-methoxyphenyl)-4,5,14,15-tetrahydro-1H-dinaphtho[2,1-e:1′,2′-g]isoindole-1,3(2H)-dione (THHBI-PhOMe). 7,12-bis(4-(diphenylamino)phenyl)-2-dodecyl-8,11-dimethoxy-4,5,14,15-tetrahydro-1H-dinaphtho[2,1-e:1′,2′-g]isoindole-1,3(2H)-dione (THHBI-PhNPh_2_).

### Steady state spectroscopy

Ultraviolet-visible (UV-vis) absorption and fluorescence spectra were performed on a UV-vis spectrophotometer (Model U-3010, Shimadzu) and a fluorescence spectrometer (F4500, Hitachi), respctively.

The fluorescence lifetime were measured by a time-correlated single-photon counting (TCSPC) spectrometer (F900, Edinburgh, UK). The samples were excited at 370 nm using a picosecond LED source (PLS-370, PicoQuant, Germany). The instrument response function (IRF) is about 400 ps.

### Time-resolved transient absorption spectroscopy

The femtosecond transient absorption investigations were performed at ~90 fs time resolution using a home-built femtosecond broadband pump-probe setup described in detail elsewhere^42^,^55^,^56^. The absorbance of the solutions was around 0.25 OD at 400 nm in a 1 mm thick quartz cuvette. The spectral data were analyzed by global analysis with the graphical interface program Glotaran based on the statistical fitting package TIMP^57^,^58^. Details of the experiment and data analysis are introduced in Supplementary Information, S1 section.

Nanosecond transient absorption measurements were carried out using a laser flash photolysis setup described previously^59^. Briefly, the setup was comprised of a Edinburgh LP920 spectrometer (Edinburgh Instruments Ltd.) combined with an Nd:YAG laser (Surelite II, Continuum Inc.). The collected spectral data were analyzed by the online software of the LP920 spectrophotometer.

### Computational details

The ground-state gas-phase geometries were optimized using density functional theory (DFT) at the B3LYP functional using basis set 6–31G*. The excited-state single point calculations have been computed with time-dependent density functional theory (TD-DFT) under the same functional and basis set. The charge difference density (CDD) cube was constructed on the basis of the optimized geometry. All calculations were carried out using Gaussian 09 package^60^.

## Additional Information

**How to cite this article**: Zhu, H. *et al*. Ultrafast Investigation of Intramolecular Charge Transfer and Solvation Dynamics of Tetrahydro[5]‐helicene-Based Imide Derivatives. *Sci. Rep*. **6**, 24313; doi: 10.1038/srep24313 (2016).

## Supplementary Material

Supplementary Information

## Figures and Tables

**Figure 1:**
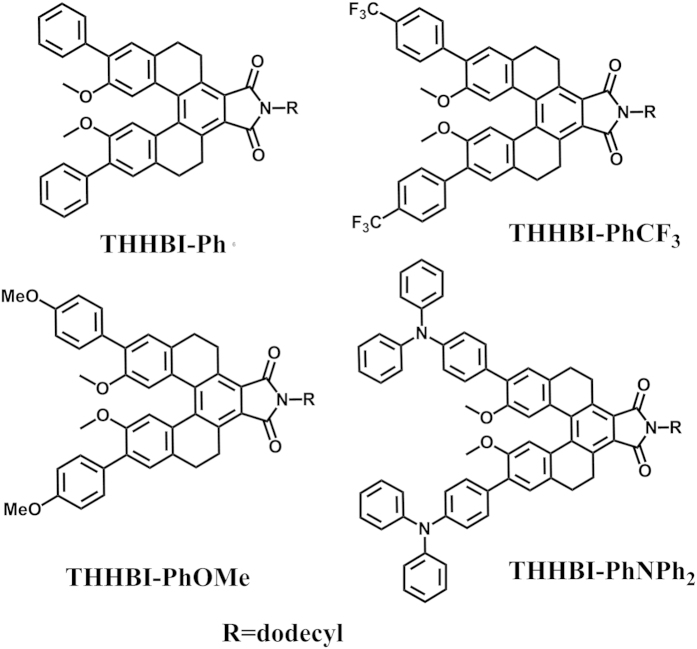
Molecular structures of the tetrahydro[5] helicene-based imide dyes.

**Table 1:** The time constants of global fitting of the femtosecond transient absorption data for all investigated dyes.

*τ*	THF			Tol		
	*τ*_*1*_	*τ*_*2*_	*τ*_*3*_	*τ*_*1*_	*τ*_*2*_	*τ*_*3*_
THHBI-Ph	0.63 ± 0.03 ps	4.3 ± 0.4 ps	5.3 ± 0.5 ns	0.76 ± 0.04 ps	7.7 ± 0.4 ps	4.2 ± 0.4 ns
THHBI-PhCF_3_	0.7 ± 0.04 ps	6.2 ± 0.5 ps	3.9 ± 0.4 ns	0.78 ± 0.05 ps	9.1 ± 0.6 ps	3.1 ± 0.3 ns
THHBI-PhOMe	0.5 ± 0.02 ps	3.3 ± 0.3 ps	6.5 ± 0.6 ns	0.58 ± 0.03 ps	6.0 ± 0.3 ps	4.8 ± 0.5 ns
THHBI-PhNPh_2_	0.4 ± 0.02 ps	2.2 ± 0.2 ps	4.8 ± 0.5 ns	0.5 ± 0.02 ps	5.2 ± 0.3 ps	5.1 ± 0.5 ns
