# Supplementary material for: Ultrafast Investigation of Intramolecular Charge Transfer and Solvation Dynamics of Tetrahydro[5]-helicene-Based Imide Derivatives
Source: Sci Rep. 2016 Apr 14;6:24313. doi: 10.1038/srep24313 (PMC4830958; doi:10.1038/srep24313)
Supplement: Supplementary Information [file srep24313-s1.pdf]

Supplementary Information

Ultrafast Investigation of Intramolecular Charge Transfer and Solvation Dynamics of Tetrahydro[5] helicene-Based Imide Derivatives

Huaning Zhu[a], Meng Li[b], Jiangpu Hu[a], Xian Wang[a], Jialong Jie[a], Qianjin Guo[a], Chuanfeng Chen*[b], Andong Xia*[a]

[a] Key Laboratory of Photochemistry, Beijing National Laboratory for Molecular Sciences (BNLMS), Institute of Chemistry, Chinese Academy of Sciences, Beijing 100190, People's Republic of China

[b] Key Laboratory of Molecular Recognition and Function, Beijing National Laboratory for Molecular Sciences (BNLMS), Institute of Chemistry, Chinese Academy of Sciences, Beijing 100190, People's Republic of China

Corresponding authors: andong@iccas.ac.cn , cchen@iccas.ac.cn

Table of Contents

S1. Details of the transient absorption spectra and data analysis.

S2. Steady-state spectral data.

S3. Quantum chemical calculation results.

S4. The fluorescence lifetime and quantum yield measurements.

S5. Femtosecond transient absorption experiments.

S6. Laser flash photolysis experiments.

S1. Details of the transient absorption spectra and data analysis.

Transient absorption spectroscopy.

Briefly, a regeneratively amplified Ti:sapphire laser (Coherent Legend Elite) produced 40 fs, 500 Hz repetition rate, 1 mJ pulses at 800 nm (FWHM: 30 nm). Doubling 90 percent of initial 800nm pulses through a 0.5 mm thick BBO (type I) crystal generated the 400 nm pump pulse. The rest of the 800 nm pulse was time-delayed by a computer-controlled optical delay line and then focused onto a 2 mm-thick water cell to produce a white light continuum (WLC), which provides a usable probe spectral range between 420 nm and 780 nm selected by a bandpass filter. The white light continuum was further split into two beams as the reference and signal beams by a broadband 50/50 beamsplitter. The pump and signal beams were overlapped in time and space on a sample cell with a 1 mm beam path length, and the reference beam passed through the unexcited part of the sample. Signal and reference beams were detected on a fiber-coupled dual-channel spectrometer (Avantes AvaSpec-2048-2-USB2). The absorbance of the solutions was around 0.25 OD at 400 nm in a 1 mm thick quartz cuvette.

Data analysis.

The differential absorbance ΔA (t, λ) is a superposition of different spectral components $\varepsilon_i(\lambda)$ weighted by their concentrations $c_i(t)$ given by the equation:

$$\Delta \mathrm{A}(t,\lambda)=\sum_{l=1}^{n} c_i(t)\varepsilon_i(\lambda) \qquad (1)$$

The global analysis usually contains two models: the parallel and sequential kinetic schemes. In this work, the sequential kinetic model was used, which produced the evolution-associated difference spectra (EADS) and could be described as:

$$\Delta \mathrm{A}(t,\lambda)=\sum_{l=1}^{n_{comp}} c_l^{EADS}(t)\,\mathrm{EADS}_l(\lambda) \qquad (2)$$

with $\mathrm{c}_i^{EADS}(t)=\sum_{j=1}^{i} b_{ji}\exp(-kjt)\oplus i(t)$ and $b_{ji}=\prod_{m=1}^{i} k_m \,/ \prod_{n=1,n\neq j}^{i}(k_n-k_j)$

where $i(t)$ is instrument response function (IRF), k_j is the decay rate of component j and the amplitudes b_{ji} of the exponential decays are defined for $j \leq i$ assuming b_{11} =1. The EADS with successively increasing lifetimes represents the spectral evolution of the excited-state species.

S2. Steady-state spectral data.

Table S1. Solvent Parameters and Stokes Shifts of THHBI-Ph, THHBI-$PhCF_3$, THHBI-PhOMe and THHBI-$PhNPh_2$.

Solvent	Parameters [a]		Stokes shift (cm^{-1})			
	$\Delta f(\varepsilon,n^2)$	E_T^N	THHBI-Ph	THHBI-$PhCF_3$	THHBI-PhOMe	THHBI-$PhNPh_2$
CHX	-0.0033	0.006	4576.7	4232.8	4921.4	4850.1
Tol	0.0159	0.099	5033.3	4604.3	5495.1	5802.8
THF	0.2103	0.207	5796.8	5129.3	6241.9	8085.8
DCM	0.2182	0.309	6172.0	5728.1	6980.3	
ACE	0.2851	0.355	6366.0	5865.9	7113.2	
ACN	0.3054	0.46	6722.7	6246.7	7892.8	

[a] Parameters are taken from ref 2. *ε* and *n* are the dielectric constant and refractive index. E_T^N are solvent polarity (expressed in the form of the Reichhardt parameters).

S3. Quantum chemical calculation results.

Table S2. The calculated frontier orbitals (HOMO and LUMO) and Charge Difference Density (CDD) for all dyes obtained by quantum chemical calculations. Green and red color correspond to the different phases of the molecular wave functions of the HOMO and LUMO, respectively. For CDD: Red color represents electron density while blue color represents hole density.

	HOMO	LUMO	CDD
THHBI-Ph	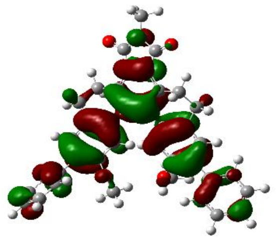	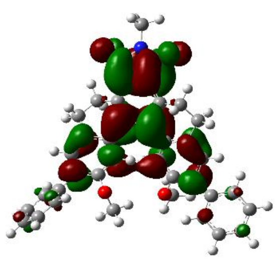	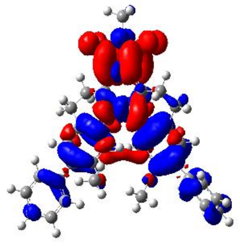
THHBI-$PhCF_3$	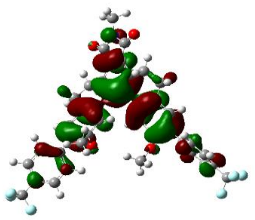	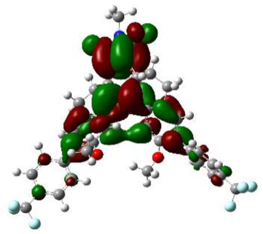	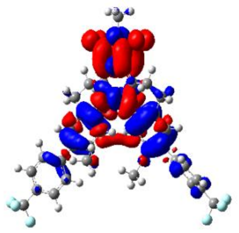

THHBI-PhOMe	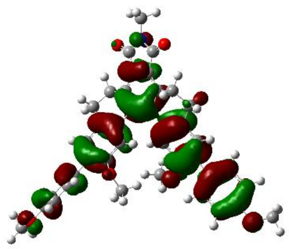	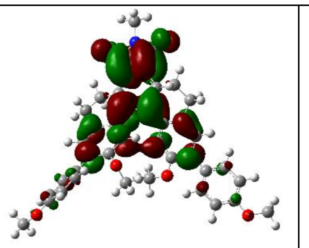	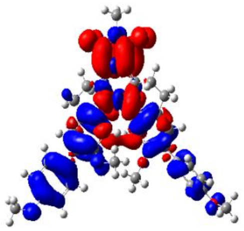
THHBI-PhNPh$_2$	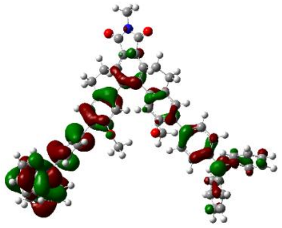	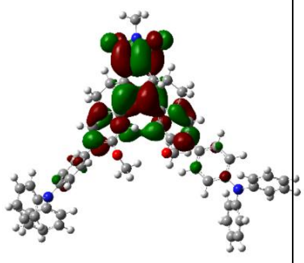	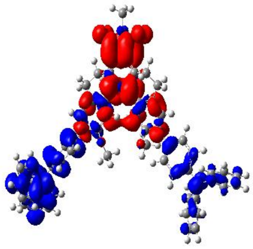

Table S3. Calculated Energies, Energy Gaps of HOMO/LUMO in the gas phase for all dyes.

	HOMO(eV)	LUMO(eV)	E_g^1 (eV)	E_g^2(eV)	E_g^3(eV)	C-C(Å)[a]
THHBI-Ph	-5.49	-2.39	3.10	3.10	3.27	1.48508
THHBI-PhCF$_3$	-6.12	-3.01	3.11	3.13	3.28	1.48533
THHBI-PhOMe	-5.21	-2.30	2.91	3.03	3.22	1.48316
THHBI-PhNPh$_2$	-4.89	-2.33	2.56	2.97	3.06	1.48203

E_g^1, energy gap of HOMO – LUMO; E_g^2, energy gap of the first excited state from theoretical calculations; E_g^3, energy gap of steady-state absorption in cyclohexane.

[a] the lengths of C-C bond linked the donor and acceptor for all dyes.

Table S4. The Onsager Cavity Radii, Slopes and Changes of the Dipole Moments of All Investigated Dyes.

	Onsager cavity radius (Å)	Slope (cm^{-1})	$\Delta\mu_{eg}$ (Debye)	μ_g (Debye)	μ_e (Debye)
THHBI-Ph	6.55	6028±700	13.0	4.1	17.1
THHBI-PhCF$_3$	6.74	5591±900	13.0	2.9	15.9
THHBI-PhOMe	6.70	7851±1300	15.3	5.1	20.4
THHBI-PhNPh$_2$	7.56	13762±2900	24.3	5.0	29.3

The Onsager cavity radii, μ_g and μ_e are obtained from the quantum chemical calculations. The slopes are acquired from the L-M plots. The $\Delta\mu_{eg}$ is calculated by $Slope = \frac{2}{hca^3}(\Delta\mu_{eg})^2$.

S4. The fluorescence lifetime and quantum yield measurements.

The fluorescence lifetime and quantum yield of all dyes in different solvents are shown in Table S5 and S6, respectively. Fluorescence decay profiles and fitted traces are displayed in Figure S1.

Table S5. The fluorescence lifetime of all dyes in different solvents obtained by TCSPC. (Unit: ns)

	$\Delta f(\varepsilon,n^2)$	η(25 ℃)	THHBI-Ph	THHBI-PhCF$_3$	THHBI-PhOMe	THHBI-PhNPh$_2$
CHX	-0.0033	0.894	3.22	3.02	3.98	4.40
Tol	0.0159	0.560	4.35	3.42	5.74	5.57
THF	0.2103	0.456	5.7	4.16	7.31	5.06
DCM	0.2182	0.413	7.47	4.67	8.98	1.22
ACE	0.2851	0.306	8.06	5.80	8.47	
ACN	0.3054	0.369	9.34	7.42	7.57	

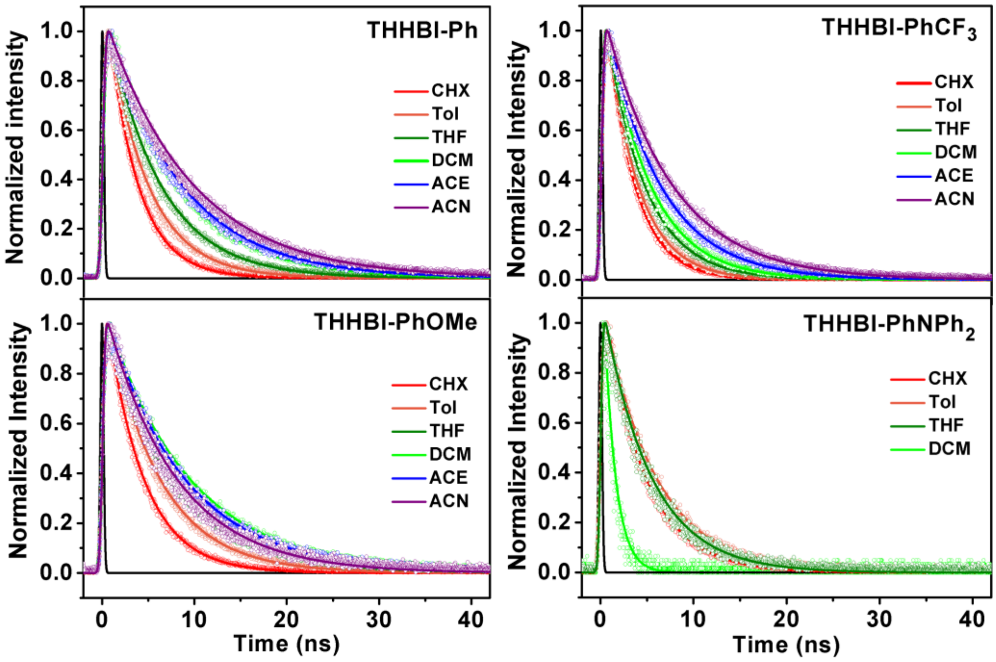

Figure S1. Fluorescence decay profiles (empty circle) and fitted traces (solid line) of all dyes in different solvents excited at 370 nm measured by TCSPC. The black line is the instrument response function (IRF).

Table S6. The fluorescence quantum yield of the dyes in different solvents.

	$\Delta f(\varepsilon,n^2)$	ε	η(25 ℃)	THHBI-Ph	THHBI-PhCF$_3$	THHBI-PhOMe	THHBI-PhNPh$_2$
[a] CHX	-0.0033	2.02	0.894	0.435	0.393	0.637	0.766
[a] Tol	0.0159	2.38	0.56	0.529	0.439	0.760	0.685
[a] THF	0.2103	7.58	0.456	0.601	0.608	0.903	0.439
[a] DCM	0.2182	8.93	0.413	0.703	0.463	0.851	0.071
ACE	0.2851	20.56	0.306	0.551	0.459	0.574	
ACN	0.3054	35.94	0.369	0.561	0.470	0.458	

[a] data are taken from ref [1]

S5. Femtosecond transient absorption spectra.

Excited state dynamics of THHBI-Ph

The femtosecond transient absorption (TA) spectra of THHBI-Ph at different time delays are depicted in Fig. S2. In THF (Fig. S2a), a broad excited state absorption (ESA) band with two maximums around 680 nm and 570 nm is observed superimposed with the stimulated emission (SE) dip around 490 nm. At a time delay to 4 ps, the 570 nm peak shifts to 560 nm and the 680 nm peak slightly blue shifts to 675 nm. The SE band rises with slight red shift, which is due to the overlapping with the blue shift of ESA band (Fig. S4d). In Tol (Fig. S2c), the TA spectral shape is similar to that in THF except the SE peak at 475 nm which is accord with its emission spectrum. Different with THF, the blue shift of ESA and rise of SE are not apparently shown. In the two solutions little change is observed in the spectral shape but intensity decay until 1ns. To figure out the relaxation mechanism, the single-value decomposition (SVD) and global fitting with a sequential model (Fig. 5a) give three time constants (Table 1) and the evolution-associated difference spectra (EADS) are shown in Fig. S2b and S2d. The fast components within 1 ps (THF: 0.63 ps, Tol: 0.76 ps) are ascribed to the conversion from initially populated Frank-Condon (FC) state to S_1 (ICT). The second one correspond to the ICT-ICT' state, and the slow components are the decay of lowest state.

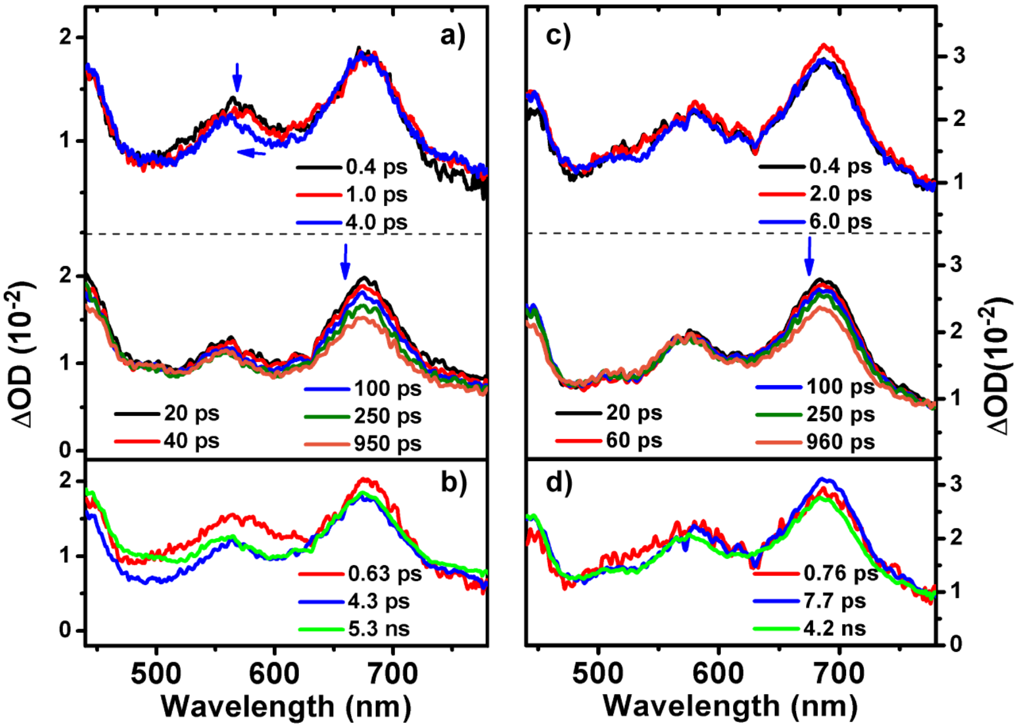

Figure S2. Femtosecond transient absorption spectra of THHBI-Ph in THF (a) and Tol (c) after 400 nm excitation at different time delays, respectively. (b) and (d) are the EADSs of THHBI-Ph obtained through the global fitting in

THF and Tol, respectively. Kinetics at selected single wavelengths for showing the quality of global fitting are shown in Fig. S5a.

In more polar THF, the ESA is blue shifted and SE is slight red shifted, which are not clearly shown but little spectral narrow in Tol. Due to the ICT characteristic，THHBI-Ph molecules interact more significantly with the polar solvent molecules than with apolar ones. Therefore, the solvation interaction leads to faster excited state relaxation (4.3 ps) in THF than in Tol (7.7 ps). However, the time constants from ICT to ICT' are longer than the corresponding solvation time of both THF (0.94 ps)[2] and Tol (2.4 ps)[3] , thus, this process are including solvation, vibrational cooling and structural relaxation. To test the relaxation pathway of the S_1 state and interpret the abnormal phenomenon of quantum yield, laser flash photolysis experiments were further performed for THHBI-Ph in THF and Tol with different gas conditions (Fig. S8 and S9). The experiments in both solutions show that the intersystem crossing (ISC) happens between the lowest excited state and triplet state. The lifetimes of triplet state in different conditions are list in Table S8. The formation of the triplet state is the crucial reason affecting fluorescence quantum yield and lifetime.

Excited state dynamics of THHBI-PhCF$_3$

The femtosecond TA spectra of THHBI-PhCF$_3$ recorded in THF are shown in Fig. S3a. A broad ESA band are shown with two peaks around 610 nm and 680 nm. And the dip at about 520 nm is assigned to the SE band. With increasing time delays, the 680 nm peak is slight blue shifted from 680 nm to 675 nm and the middle ESA shift from 610 nm to 600 nm. In Tol (Fig. S3c), the spectral shape looks like the same as that in THF. The spectra show slight shift of ESA and SE and little spectral narrow of ESA. The TA data were also analyzed through SVD and global fitting with a sequential model. The results provide three time constants for best fitting (Table 1) and the resulting EADSs are displayed in Fig. S3b, S3d. The three process are assigned to the FC-ICT, ICT-ICT' and the decay of the S_1 state, respectively. The second components are 6.2 ps and 9.1 ps for THF and Tol, respectively, which are quite different from the solvation time of these two solvents. The steady-state spectra and theoretical calculations reveal very weak ICT characteristic in THHBI-PhCF$_3$ and the TA spectra also show little blue shift of ESA, which suggests the solutes have very weak interaction with the THF and Tol solvent molecules. Thus, this process mainly comes from the vibrational relaxation of S_1 state accompanying with structural relaxation and part of solvation. Additionally, the laser flash photolysis measurements of the THHBI-PhCF$_3$ in different solvents confirm the formation of triplet

state which has influence on the fluorescence quantum yield. The triplet state decays with a single time constant of 0.09 us (0.1 us) in oxygenated THF (Tol) solution but exhibits dramatic long lifetime of 58 us (51.8 us) in N_2-purged THF (Tol) (Table S8). The nanosecond transient absorption spectra and kinetics are present in Fig. S8 and S10.

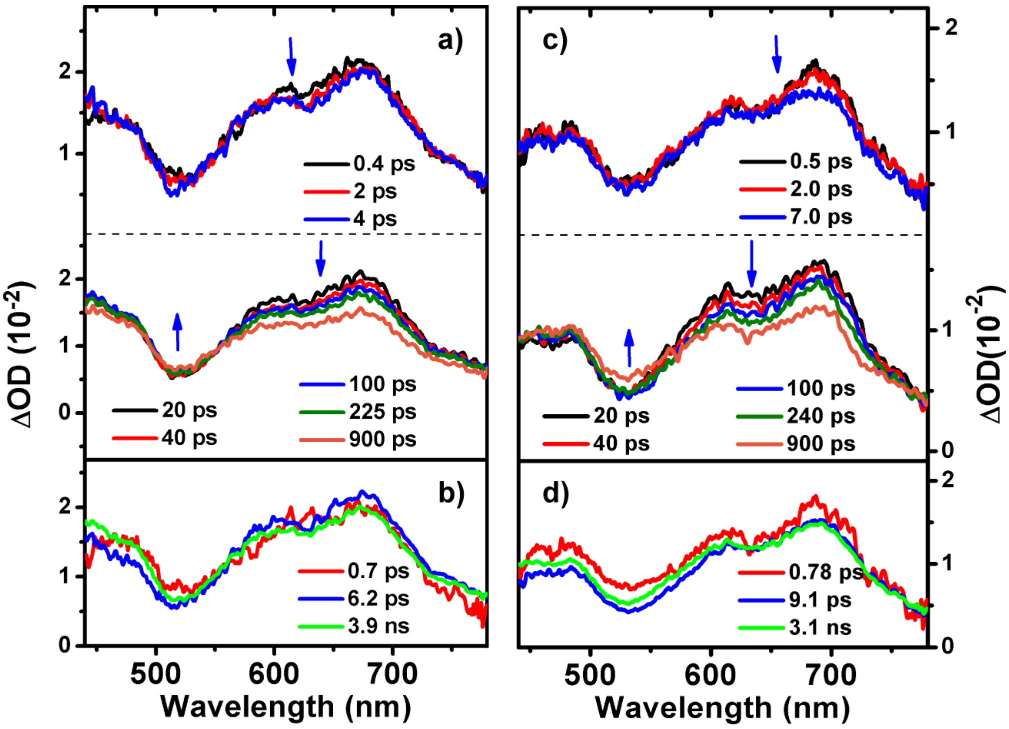

Figure S3. Femtosecond transient absorption spectra of THHBI-PhCF_3 in THF (a) and Tol (c) after 400 nm excitation at different time delays, respectively. (b) and (d) are the EADSs of THHBI-PhCF_3 obtained through the global fitting in THF and Tol, respectively. Kinetics at selected single wavelengths for showing the quality of global fitting are shown in Fig. S6b.

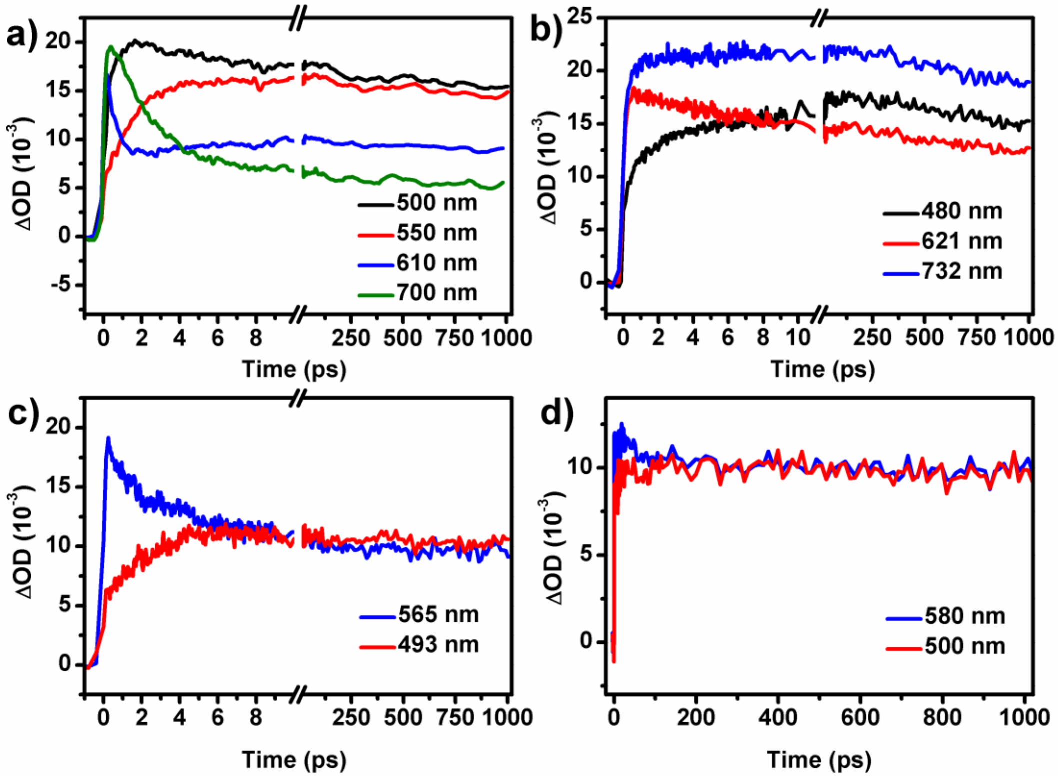

Figure S4. The kinetic traces of the compounds at different wavelengths. (a) and (b) are for THHBI-PhNPh$_2$ in THF and Tol, respectively. (c) and (d) are for THHBI-PhOMe and THHBI-Ph in THF solution, respectively.

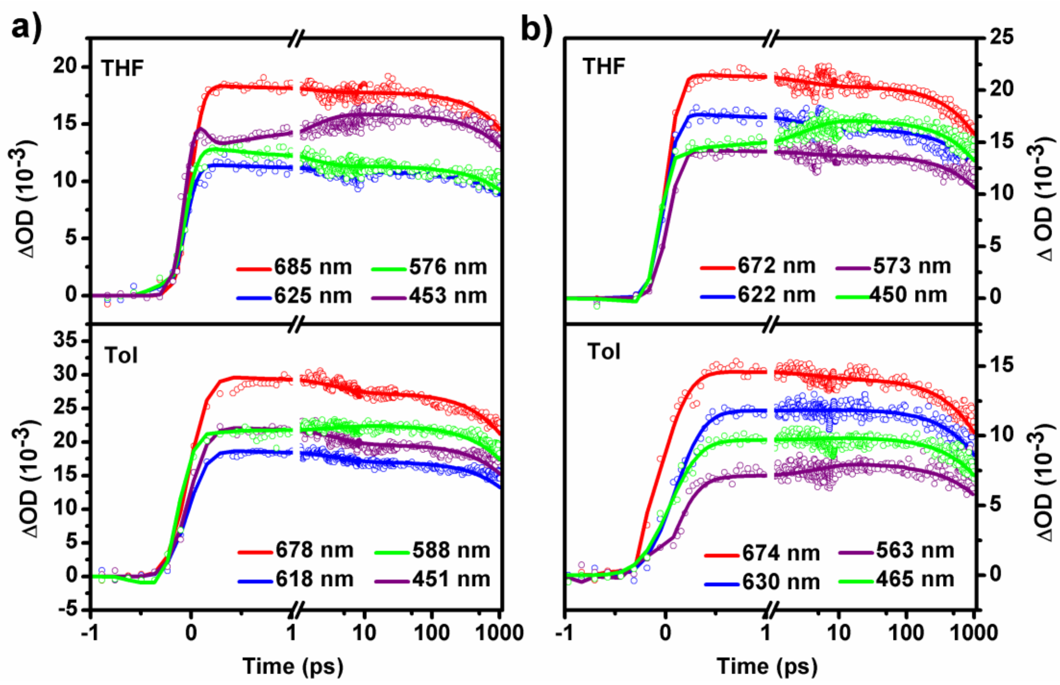

Figure S5. The kinetics at selected wavelengths of THHBI-Ph (a) and THHBI-PhCF$_3$ (b) for showing the quality of global fitting.

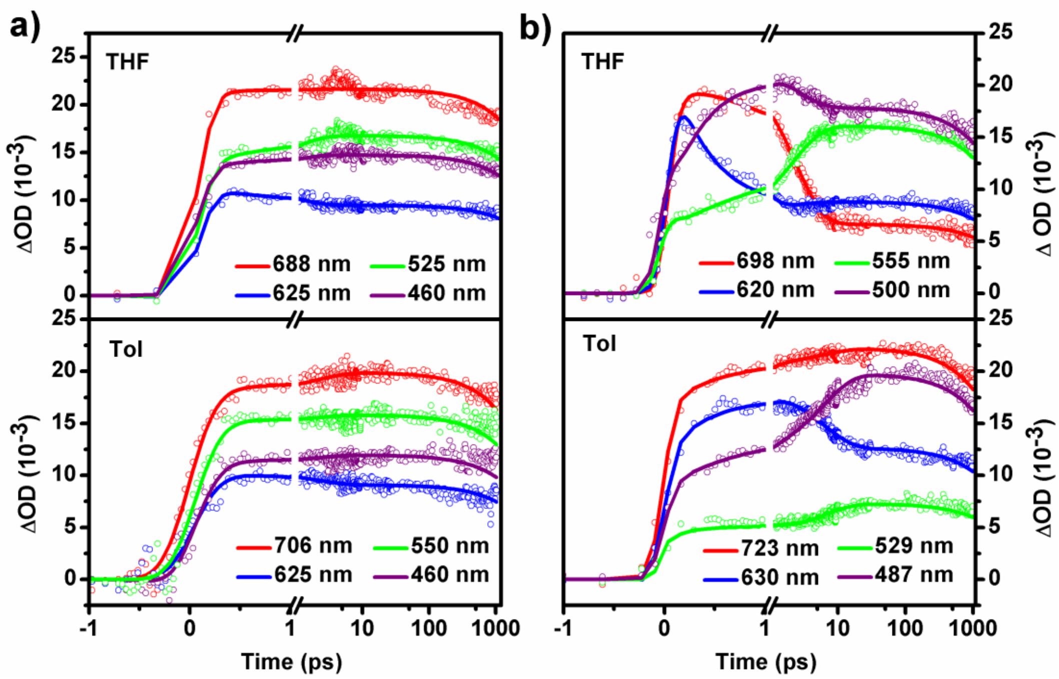

Figure S6. The kinetics at selected wavelengths of THHBI-PhOMe (a) and THHBI-PhNPh$_2$ (b) for showing the quality of global fitting.

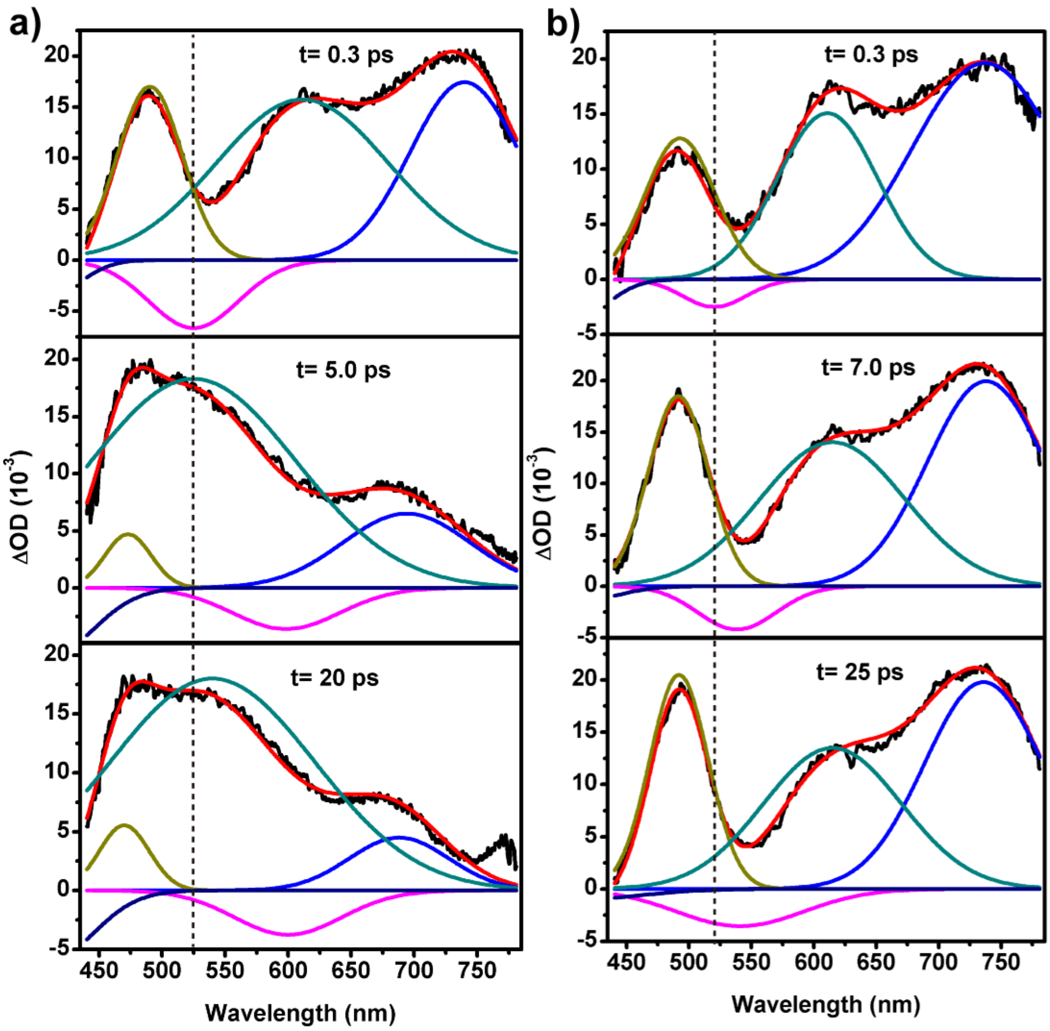

Figure S7. Broad band transient absorption peaks of THHBI-$PhNPh_2$ were fitted using Gaussian peaks. Selected transient spectra (black line) and fits (red line) of THHBI-$PhNPh_2$ in THF (a), Tol (b), at three exampled decay time, respectively. Dashed lines are guides to the eye for identifying the spectral shift.

Table S7. Fitting Parameters for the Peak Shift Correlation Function of THHBI-$PhNPh_2$ in THF and Tol versus Solvent Parameters.

	THF	Tol
Δf	0.21	0.0159
τ_1 (ps)	0.22 (35%)	0.37 (79%)
τ_2 (ps)	1.11 (65%)	5.56 (21%)
$\langle\tau\rangle$ (ps)	0.8	1.45
$\langle\tau\rangle$* (ps)	0.94	2.4

Δf is the polarity index. All correlation functions are fitted by two exponential decays: $C(t) = a_1 \exp(-t/\tau_1) + a_2 \exp(-t/\tau_2)$. <τ>*is the average solvation time. The average solvation time is calculated by $\langle\tau\rangle = (a_1\tau_1 + a_2\tau_2)/(a_1 + a_2)$.

S6. Laser flash photolysis spectra.

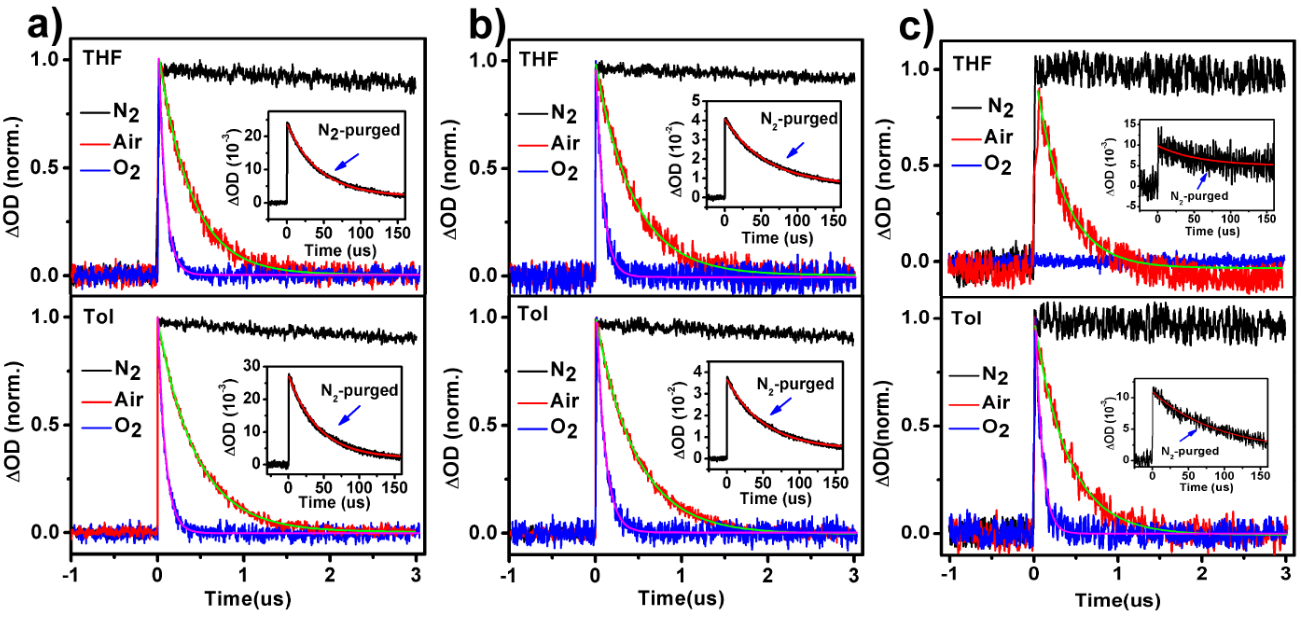

Figure S8. The kinetic traces of THHBI-Ph (a), THHBI-$PhCF_3$ (b) and THHBI-PhOMe (c) in THF and Tol solutions with different gas conditions (N_2, Air, O_2).

THHBI-Ph

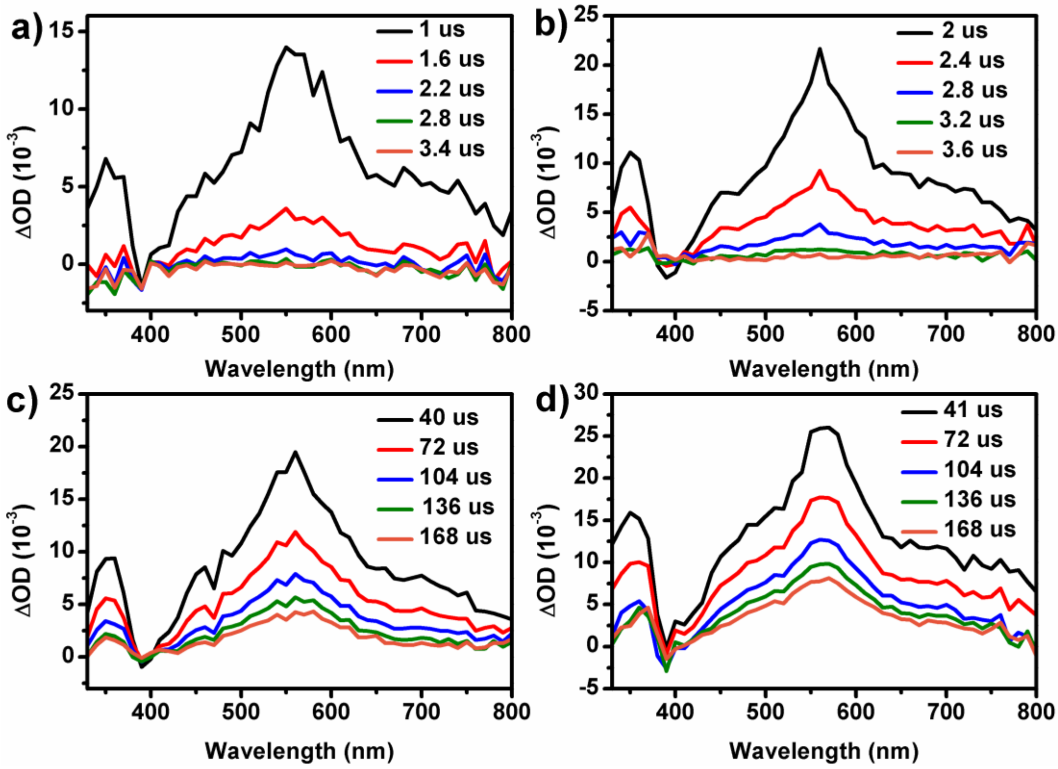

Figure S9. The nanosecond transient absorption spectra of THHBI-Ph in THF (a, c) and Tol (b, d). a and b are the spectra in the air condition, c and d are the spectra in N_2 purged solutions.

THHBI-PhCF$_3$

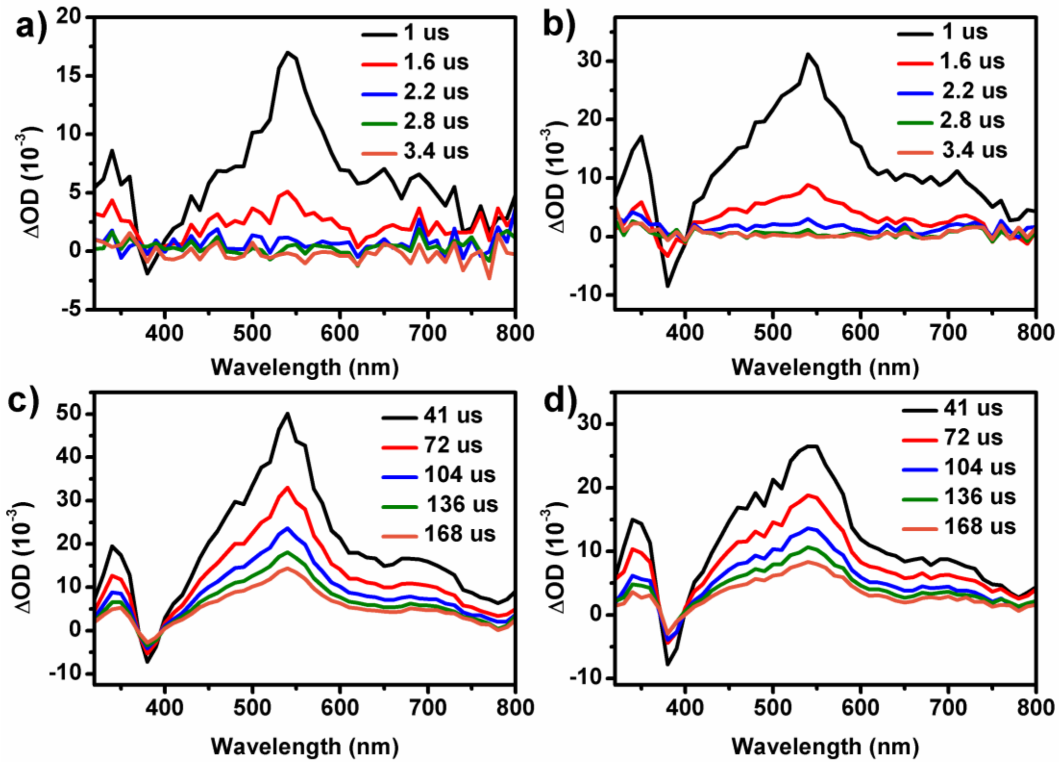

Figure S10. The nanosecond transient absorption spectra of THHBI-PhCF$_3$ in THF (a, c) and Tol (b, d). a and b are the spectra in the air condition, c and d are the spectra in N_2 purged solutions.

THHBI-PhOMe and THHBI-PhNPh$_2$

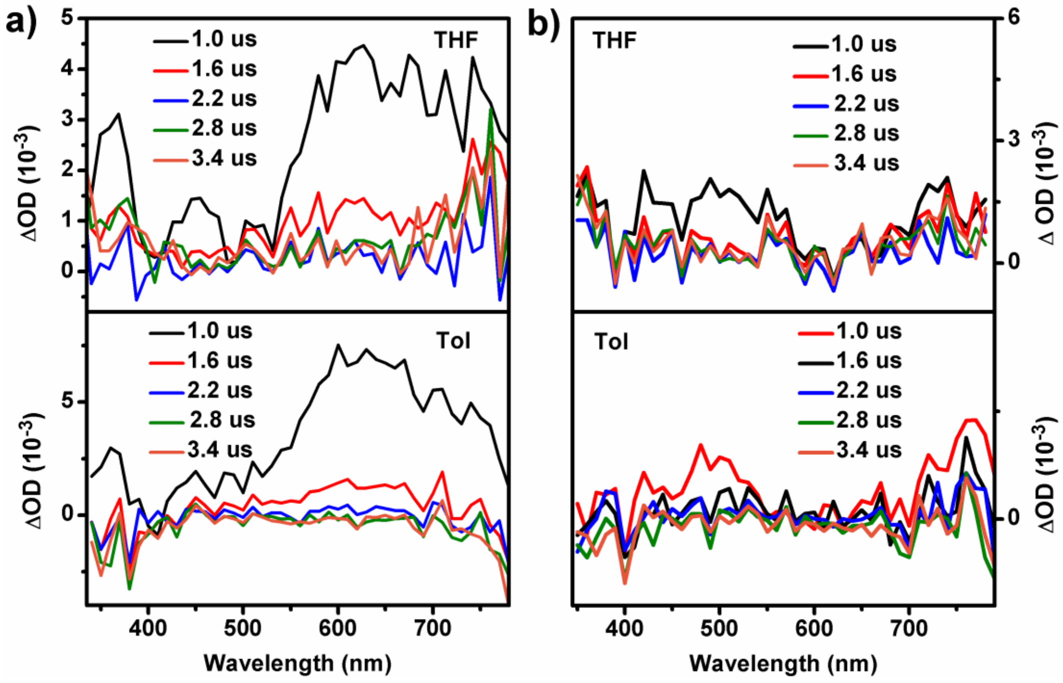

Figure S11. The nanosecond transient absorption spectra of THHBI-PhOMe (a) and THHBI-PhNPh$_2$ (b) in THF (Tol) in air condition.

Table S8. The lifetime of the triplet state in different gas conditions obtained by laser flash photolysis experiments. (Unit: us)

	Tol			THF		
Dye	O_2	Air	N_2	O_2	Air	N_2
THHBI-Ph	0.09	0.44	42.35	0.08	0.38	43.45
THHBI-PhCF_3	0.10	0.46	51.80	0.09	0.44	58.01
THHBI-PhOMe	0.08	0.40	90.45		0.35	58.05
THHBI-PhNPh_2						

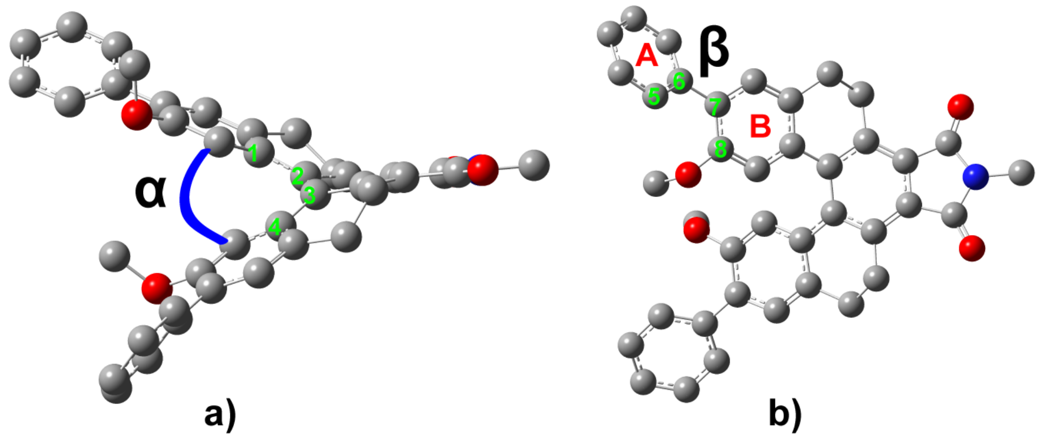

Figure S12. The optimized structures of the dyes. *α* and *β* are the dihedral angle of the C1-C2-C3-C4 (torsion angle of the helicene-based moiety) and C5-C6-C7-C8 (between the aromatic rings A and B).

Table S9. The dihedral angle of the optimized ground and excited state structures (shown in Fig. S12) for all investigated dyes.

	Ground state		Excited state	
	α	*β*	*α*	*β*
THHBI-Ph	38.8	42.2	50.8	35.5
THHBI-PhCF_3	39.7	43.3	51.2	35.3
THHBI-PhOMe	39.3	39.7	50.7	34.9
THHBI-PhNPh_2	38.9	38.4	47.8	34.5

References

1 Li, M. *et al.* Tetrahydro[5]helicene-based imide dyes with intense fluorescence in both solution and solid state. *Chem. Commun.* **50**, 2993 (2014).

2 Horng, M. L., Gardecki, J. A., Papazyan, A. & Maroncelli, M. Subpicosecond measurements of polar solvation dynamics: Coumarin 153 revisited. *J. Phys. Chem.* **99**, 17311-17337 (1995).

3 Larsen, D. S., Ohta, K. & Fleming, G. R. Three pulse photon echo studies of nondipolar solvation: Comparison with a viscoelastic model. *J. Chem. Phys.* **111**, 8970-8979 (1999).
